# Supplementary material for: Transcriptional Analysis of the Differences between ToLCNDV-India and ToLCNDV-ES Leading to Contrary Symptom Development in Cucumber
Source: Int J Mol Sci. 2023 Jan 22;24(3):2181. doi: 10.3390/ijms24032181 (PMC9916722; doi:10.3390/ijms24032181)
Supplement: Supplementary file 1 [file ijms-24-02181-s001.zip › 221219 Supplementary data.pdf]

Supplementary

Table S1: Representative DEGs associated with two ToLCNDV infected condition

Isolates	ID	log2FoldChange	padj	Gene function
ToLCNDV-ES	CsaV3_3G028410.1	2.6840803	1.94E-19	subtilisin-like protease SBT1
	CsaV3_6G046540.1	2.1647143	0.0046005	Carboxyl methyltransferase
	CsaV3_2G036090.1	2.052104	1.95E-13	4-coumarate-CoA ligase
	CsaV3_6G007460.1	1.9428606	0.0024314	Protein JASON like
	CsaV3_6G049170.1	1.8784914	1.37E-14	glucose-6-phosphate/phosphate translocator 2
	CsaV3_3G003660.1	-3.557204	0.0007748	(-)-germacrene D synthase-like
	CsaV3_3G049550.1	-3.697095	0.0004389	expansin-like B1
	CsaV3_2G022180.1	-3.732506	0.0078682	NAC domain protein
	CsaV3_7G026200.1	-3.827982	1.53E-05	Pectinesterase
	CsaV3_3G041400.1	-4.642323	0.004399	Ribonuclease (RNase LC1)
ToLCNDV-India	CsaV3_6G050450.1	8.6826956	4.91E-29	NAC domain-containing protein
	CsaV3_2G031820.1	8.0244992	1.37E-07	Boron transporter like
	CsaV3_6G038040.1	7.4878828	1.71E-17	Xyloglucan endotransglucosylase/hydrolase
	CsaV3_6G012270.1	6.6322132	3.11E-05	Calcium-binding protein pbp1
	CsaV3_1G038400.1	6.4463479	4.34E-16	calmodulin-binding protein 60 C-like isoform X2
	CsaV3_2G017880.1	-6.470612	2.49E-84	terpene synthase 10-like
	CsaV3_3G003660.1	-6.599902	6.37E-10	(-)-germacrene D synthase-like
	CsaV3_6G046510.1	-6.62698	4.70E-12	salicylate carboxymethyltransferase-like
	CsaV3_3G033740.1	-6.953546	2.85E-44	2-oxoglutarate (2OG) and Fe(II)-dependent oxygenase superfamily

	CsaV3_6G040200.1	-9.365912	1.87E-42	Threonine dehydratase

Table S2: Primer sets used in validation by qRT-PCR

Gene name	Primer name	Sequence (5'-3')
CsaV3_7G001540	qRT-1-F	CCTTTTCTCCTCAGCCACTG
	qRT-1-R	AAGATCGCCATAGCCACAAG
CsaV3_7G004500	qRT-2-F	CTGACGACGAAGACGAACAA
	qRT-2-R	GCGGAGTCTTGACTCTACCG
CsaV3_6G038040	qRT-3-F	GGTCCACGTGCCAAGATACT
	qRT-3-R	CAGAGTTTCCAGGGACAAGC
CsaV3_5G006290	qRT-4-F	GTTTGTTCGGCGATACCACT
	qRT-4-R	GATTCCGCGTCTTTGAATGT
CsaV3_5G031640	qRT-5-F	CGTGTCTCAACGGATCAATG
	qRT-5-R	ACCAAACCAGCACCAGAAAC
CsaV3_4G023890	qRT-6-F	GTTTTGGGAAGATTGGAGCA
	qRT-6-R	GAAGATCCTTCGGTGGTCAA
CsaV3_2G017880	qRT-7-F	AGGCATTGGATTTGAACCTG
	qRT-7-R	TTGCTCCAATATCCCACCTC
CsaV3_4G034400	qRT-8-F	GGGATTTGCAGATTGGAGAA
	qRT-8-R	GGATTGGACAAATGGTTTGG
CsaV3_3G014180	qRT-9-F	CCTTGGCCATTGGAGTTATG
	qRT-9-R	CCTCTTGAGTAGGCCCACAA
CsaV3_3G022370	qRT-10-F	CAATCCACAGTGCGCTAGAA
	qRT-10-R	TCTCCGTCGTCTTCCTCTGT

Table S3: Candidate genes belong to flavonoid pathway/Oxidation-reduction process and their primer sets used in qRT-PCR

Gene name	Function	Primer name	Sequence (5'-3')
CsaV3_2G036090	4-coumarate-CoA ligase	qRT-flavo-1-F	CCACCCATGTTTTCCGATCC
		qRT-flavo-1-R	GGTGGGTTTCGGAGTAGGAA
CsaV3_2G007940	4-coumarate-CoA ligase	qRT-flavo-2-F	CAGCAGGTGGATGGAGAGAA
		qRT-flavo-2-R	GGAGAAGAGAGGAGACGTCG
CsaV3_3G027830	chalcone synthase	qRT-flavo-3-F	TCTATGGTTGGTCAGGCGTT
		qRT-flavo-3-R	TTCCCTCAAATGTCCGTCGA
CsaV3_4G027940	chalcone synthase	qRT-flavo-4-F	TGCACTGCTTCAACCAACTC
		qRT-flavo-4-R	AACGACAAGAACACGAGCAC
CsaV3_7G010170	Allene oxide synthase	qRT-oxre-1-F	CCACAAATTCCCCTCCCTCT
		qRT-oxre-1-R	AAGAGATGAAGGGACCTGGC
CsaV3_2G012080	9-cis epoxycarotenoid dioxygenase	qRT-oxre-2-F	CTAATCCCATGCATGCACCC
		qRT-oxre-2-R	AGGGAAAACAAGTCGGCCTA
CsaV3_4G023890	Lipoxygenase	qRT-oxre-3-F	TGCTAGGACCAACATGCTCA
		qRT-oxre-3-R	TAGCGTCCCCAATGTACTCC
CsaV3_4G023920	Lipoxygenase	qRT-oxre-4-F	TGAGCAGACCACCCATTGAT
		qRT-oxre-4-R	TTCCACGCAGCAATGAGTTC
CsaV3_4G007760	9-cis epoxycarotenoid dioxygenase	qRT-oxre-5-F	AACTCCATGGACACTCTGGG
		qRT-oxre-5-R	GCCGTCCGTTGAAATAGACC

Table S4: RNA-seq data of 9 candidate genes belong to oxidation/reduction and flavonoid pathway

	**Gene ID**	**Gene function**	**log2Foldchange**	
			**ToLCNDV-ES**	**ToLCNDV-India**
**Oxidation/reduction**	CsaV3_7G010170	Allene oxide synthase	-1.49003	-3.22332
	CsaV3_2G012080	9-cis epoxycarotenoid dioxygenase	-2.237106	-4.90913
	CsaV3_4G023890	Lipoxygenase	-1.034779	-4.08269
	CsaV3_4G023920	Lipoxygenase	-1.635543	-3.83411
	CsaV3_4G007760	9-cis epoxycarotenoid dioxygenase	-1.78984	-3.27404
**Flavonoid pathway**	CsaV3_2G036090	4-coumarate-CoA ligase	2.052104	1.267342
	CsaV3_2G007940	4-coumarate-CoA ligase	-1.03634	-2.28147
	CsaV3_3G027830	Chalcone synthase	2.949624	2.342171
	CsaV3_4G027940	Chalcone synthase	-3.43204	-1.42102
